# The Effects of Park Based Interventions on Health: The Italian Project “Moving Parks”

**DOI:** 10.3390/ijerph19042130

**Published:** 2022-02-14

**Authors:** Stefania Toselli, Laura Bragonzoni, Laura Dallolio, Grigoletto Alessia, Alice Masini, Sofia Marini, Giuseppe Barone, Erika Pinelli, Raffaele Zinno, Mario Mauro, Gerardo Astorino, Pietro Loro Pilone, Simona Galli, Pasqualino Maietta Latessa

**Affiliations:** 1 Department of Biomedical and Neuromotor Sciences, University of Bologna, 40126 Bologna, Italy; stefania.toselli@unibo.it (S.T.); laura.dallolio@unibo.it (L.D.); alice.masini7@unibo.it (A.M.); mario.mauro4@unibo.it (M.M.); 2 Department of Life Quality Studies, University of Bologna, 47921 Rimini, Italy; laura.bragonzoni4@unibo.it (L.B.); sofia.marini2@unibo.it (S.M.); giuseppe.barone8@unibo.it (G.B.); erika.pinelli2@unibo.it (E.P.); raffaele.zinno2@unibo.it (R.Z.); pasqualino.maietta@unibo.it (P.M.L.); 3 Department of Public Health, AUSL di Bologna, 40124 Bologna, Italy; gerardo.astorino@ausl.bologna.it (G.A.); p.loropilone@ausl.bologna.it (P.L.P.); simona.galli@ausl.bologna.it (S.G.)

**Keywords:** citizen health, green infrastructure, green urban space, health status, mental health, park, physical activity

## Abstract

Obesity and physical inactivity are global health problems responsible for the risk increment of noncommunicable diseases. To overcome these problems, interventions aimed at increasing physical activity (PA) are necessary. Green space can have a positive influence on promoting PA, so, the aim of the present study was to assess the effectiveness of the project “The moving parks project”, which provides for the administration of PA to citizens within Bologna’s parks (Italy). An ad hoc questionnaire was administered before and after three months of outdoor PA. A total of 329 adult subjects participated in the survey. At follow-up, all psychosocial parameters showed an improvement, with a reduction in the state of tension, sadness and fatigue, and an improvement in the state of energy, serenity, and vitality. The impact of the interventions carried out in the “Moving Parks project” was positive and appears to be a good strategy for improving health outcomes.

## 1. Introduction

Body mass index (BMI) has increased steadily in most countries, concurrently with a rise in the proportion of the population living in cities, suggesting that urbanization is one of the most important drivers of the global rise in obesity [1]. As regards Italy, Di Bonaventura et al. (2018) reported that 52.26% of the adult population were normal weight, 34.85% were overweight, and 12.89% were obese (9.49% were obese class I, 2.28% were obese class II, and 1.12% were obese class III) [2]. Obesity is a risk factor for a variety of diseases, such as cardiovascular disease, cancer, type 2 diabetes (T2D), osteoarthritis, nonalcoholic fatty liver disease, sleep apnea, and psychiatric conditions [3,4,5]. Moreover, obesity predisposes to the functional impairment of mobility. Nowadays, it is widely known that being overweight and obesity are related to unhealthy lifestyle habits such as physical inactivity and malnutrition [6,7]. Sedentary behavior (SB), defined as any waking behavior characterized by an energy expenditure ≤1.5 metabolic equivalents (METs), has increased in industrialized countries in the last decades, with the average adult spending more than half of the day on SB [7,8,9]. From 2002 to 2017 the European adult population showed an increased trend in SB prevalence, for both males and females [9,10]. To face this situation, the World Health Organization (WHO) recommends limiting the amount of time spent in SB, reducing sedentary time with physical activity (PA) of any intensity to obtain health benefits [10,11]. PA behaviors are associated with health benefits and could prevent the onset of several diseases [11,12]. In addition, participation in physical activity is useful for maintaining and slowing the physiological age-dependent decline of the musculoskeletal system, a process that leads to degenerative forms of arthrosis, as well as to a prevalent loss of strength and elasticity [13,14]. In addition, PA positively affects psychological mental health, for instance, lowering depression, reducing anxiety and stress perception, and improving mood [14,15,16]. Despite the evidence, only 31% of Italian adults (35% males and 26% females) reported having a physically active job, carried out 30 min of moderate activity at least 5 days per week or at least 20 min of intense PA at least 3 days per week in 30 days [13].

However, several factors influence participation in PA and greater attention has recently focused on the role of the environment in promoting healthy behaviors such as PA [17,18]. In such a scenario, green space may be an environment that influences the practice of PA by offering a safe, accessible and attractive place for exercise, such as walking, running, cycling or playing ball games. Indeed, it is hypothesized that those who have access to more green space in their local environment might be expected to achieve higher levels of PA [17]. For all these reasons, the World Health Organization in the document “Global action plan on physical activity 2018–2030” highlights, among the strategies to promote health and to reduce physical inactivity, the opportunity of improving PA programs and interventions in parks and other natural environments [11]. PA in a natural environment has multiple positive effects: sustaining physical health, reducing stress and anxiety, improving self esteem and mood, and promoting mental focus [19,20,21,22,23]. Open spaces, including parks and gardens, are an essential part of a network of physical and social wellbeing. In this regard, two studies on Chinese older adults confirmed that psychological wellbeing was influenced by the quality of open space, and was positively correlated with walkable environment, and, in particular, with the perceived attributes of accessibility and walking facilities [24,25]. Parks not only provide space to exercise but also allow their user to overcome mental isolation. Kothencz et al. (2017) found that perceived green space characteristics were strong predictors of wellbeing [26]. Moreover, green space was reported to enhance social connections and reduce loneliness and segregation, in Turkey [27]. Some studies highlight that the average number of minutes of PA from moderate to vigorous intensity among urban participants, was more common in green spaces rather than in other settings (school, home, street), with significant differences [28,29,30]. In addition, the beneficial effects of PA in natural environments were higher than those carried out in more synthetic environments, reducing negative emotions [15,31,32].

Improving parks’ availability and users’ satisfaction with parks may increase visitation and, consequently, increase physical activity and time spent outdoors [33]. There are different strategies to increase the practice of outdoor PA: changing the physical structure of the parks (i.e., adding walking trails) to facilitate physical activity (place level interventions) and/or providing free or low cost group wellness programs in parks (person level intervention) [34]. Place based interventions were more common than person based interventions [35]. Only a few studies have evaluated person based interventions in nonclinical populations. These studies were generally numerically limited and included walks in the park. In Singapore, Petrunoff et al. (2021) and Müller-Riemenschneider et al. (2020) found that a supervised Park Prescription intervention of PA effectively increased recreational PA, park use, park PA, and psychological quality of life [36,37]. Sellers et al. (2012) showed that a 30-min self timed brisk walk taken in a park compared with one taken in an urban setting in Glasgow, Scotland, can impact more healthy adults’ capacity to perform health enhancing PA, facilitating the achievement of brisk walking bouts of ≥10 min in duration [38]. Furthermore, de Bloom et al. (2017) considered the effects of park walking and relaxation exercises during the lunch breaks of Finnish workers [39]. The most consistent positive effects throughout the day were reported by the park walking group. Despite the beneficial effects of PA practiced in green spaces, no studies related to this aspect that concern Italy have been conducted.

Thus, there is a strong interest in designing interventions aimed at increasing physical activity in Italian adults and understanding their effects. In addition, Good Health and Wellbeing is one of the UNESCO Sustainable Development Goals [40]. For this reason, the project “The moving parks project”, was conceptualized to address research evidence gaps and evaluate the effectiveness of a structured PA in the park, carefully developed and supervised by qualified instructors on (1) PA behaviors (intention to practice PA, importance of PA); (2) weight status and psychological general wellbeing.

“The moving parks project” aimed to give all citizens the opportunity to become familiar with the public green areas in the municipal territory and to integrate motor experience with health and wellness education activities. We expect an increase in levels of PA, and an improvement i weight status and of the psychosocial wellbeing in project participants.

## 2. Materials and Methods

### 2.1. The Project

**Figure A1:**
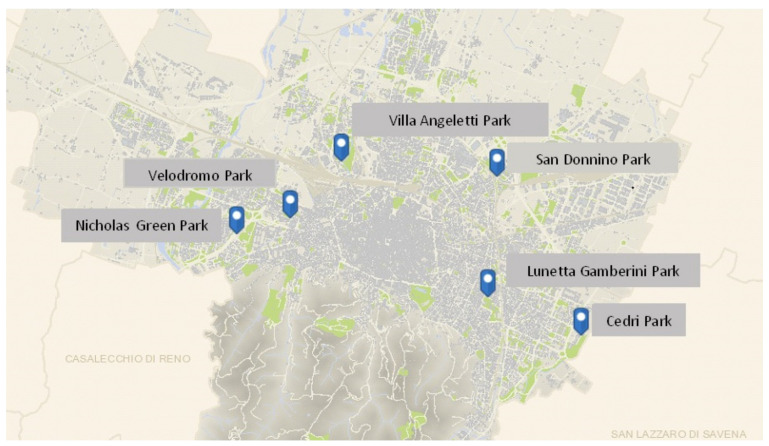
Map of Bologna, with highlighted the six parks considered.

Bologna has a green public area that is around 1000 hectares, equal to about the 7% of the municipal territory. In the city, there are around 250 spaces, between parks and gardens [41]. “The moving parks project” was a project created by the Municipality of Bologna in collaboration with the Department of Public Health of the Bologna Local Health Authority in 2010, with the goal to spread the importance of outdoor physical activity. The aim was also to increase the quality of life of all citizens through the regular practice of physical activity and contact with the natural environment. This project was carried out for three months during the summer period and involved six municipal parks. The selected parks were chosen, each in a different neighborhood of the city (Appendix A Figure A1). In addition, the project involved fourteen sports associations offering different types of activities, such as Nordic walking, pilates, tai chi, postural training. All activities were free of charge. Every activity was proposed twice a week and managed by qualified instructors. The study involved the administration of a specific questionnaire before and after the physical activity.

In order to enroll participants in “The moving parks project”, strategies of distribution fliers were adopted and many local commercial activities, such as a pharmacy, market, clinic, etc., were involved. In addition, a specific webpage, at “www.comunedibologna.it (18 November 2021)”, was made to promote and achieve larger adhesion.

### 2.2. Questionnaire

The questionnaire was created ad hoc to investigate the participants’ habits and their health status, before and after the three months of outdoor physical activity. The questionnaire was divided into three parts: (1) general information about the participants, (2) information about physical activity, and (3) psychological general wellbeing. The first part asked for general information, such as age range (18–44; 45–64, >65), weight, height, neighborhood, means of transport and whether participants usually used stairs or lift. The second part asked about the importance of physical activity on a scale from 0 to 100, in which 0 meant nothing and 100 extremely important. In addition, there were questions about future intentions to exercise once the project is completed. The third part investigated participants’ psychological amd general wellbeing, and health related quality of life through a previously developed and validated Psychological General Well Being Index short form questionnaire version (PGWB-S) [42]. The PGWBI integral version includes six domains composed of 22 items: anxiety (items 5, 8, 17, 19, 22), depressed mood (3, 7, 11), positive wellbeing (1, 9, 15, 20), self control (4, 14, 18), general health (2, 10, 13), and vitality (6, 12, 16, 21) [43]. The original scoring by items was 0–5 with a maximum score of 110, or was 1–6 with a score range of 22–132.

To validate the presented short version, some authors assessed a multiple stepwise regression procedure and selected the minimum number of items that explained at least 90% of the variance of the original questionnaire [42]. In addition, a previous study showed that many PGWBI items could be correctly described by others, which are most highly correlated (Pearson moment correlation) [43]. According to these results, the following short version could well outline information about five of the original six domains (anxiety, depress mood, positive wellbeing, self control, and vitality). The PGWB-S presents only six items of the 22 (5, 6, 7, 18, 20, 21) with score ranging from 1 to 6, where 1 means poorest QoL and 6 means best QoL. In particular, the questions were: in the last four weeks, (1) did you feel full of energy?; (2) did you feel nervous?; (3) did you feel downhearted and blue?; (4) did you feel calm and peaceful?; (5) did you feel happy?; and (6) did you feel worn or tired? At the beginning, the possible answers were “none of the time”, “a little bit of the time”, “some of the time”, “a good bit of the time”, “most of the time” and “all of the time”. Then, each response was transformed into a discrete observation to obtain only items with a Likert scale.

The questionnaires were administered both on paper and as an online survey using Google Moduli Form. Several trained instructors taught participants how to fill out on paper questionnaire and each participant could opt to complete it on paper or online. Two global social networks were used to promote people participation (Facebook^®^, Meta Platforms, Inc, Cambridge, MA, USA; LinkedIn^®^, Microsoft, Sunnyvale, CA, USA). All participants were informed and gave us privacy consent to handle their personal data. They could fill out the survey with no Google sign in request. They could manually enter all general information or allow the social networks to complete them. The questionnaire was self administered in the Italian language. Each completed survey was saved on a Google database, and we gathered all data as an Excel spreadsheet (Microsoft Office^®^, Microsoft Corporation, Redmond, WA, USA). The survey was approved by the bioethics Committee of the University of Bologna (prot. N 169182).

### 2.3. Statistical Analysis

The data analysis was performed using Statistica for Windows, version 8.0 (Stat Soft Italia SRL, Vigonza, Padua, Italy). To test the questionnaire’s reliability, its dimension was evaluated by a confirmatory factory analysis (CFA), and its internal consistency was calculated by the Cronbach’s alpha coefficient on the PGBW-S items. In order to perform the CFA, six items were selected (PGWB-S), ranging from 1 to 6 as a Likert scale. To report the model fit statistic, the comparative fit index (CFI) and the Tucker–Lewis index (TLI) were calculated. Both CFI and TLI values ranged from 0 to 1, with higher values indicating better fit [44]. In addition, the root mean square residual (SRMR) value was calculated, ranging from 0 to 1, where lower value is indicative of an acceptable model. According to conventional criteria, the CFI ≥ 0.90, SRMR ≥ 0.10 and TLI ≥ 0.90 indicated an acceptable fit [45,46]. To estimate how much this model explains PGBWI variability, the total R^2^ and for each variable were calculated. Finally, Cronbach’s alpha was considered reliable for values between 0.5 and 0.9.

The means ± SD data from baseline to follow-up were calculated. Variable’s normality was verified with the Shapiro–Wilk test. Paired samples Wilcoxon test were carried out to value the differences between the two measurements. Percentage frequency was determined for qualitative variables (weight status) and the differences in the frequencies were tested by the chi-squared test. The results were considered statistically significant if the value was lower than 0.05.

## 3. Results

### 3.1. Sample Size

**Figure 1:**
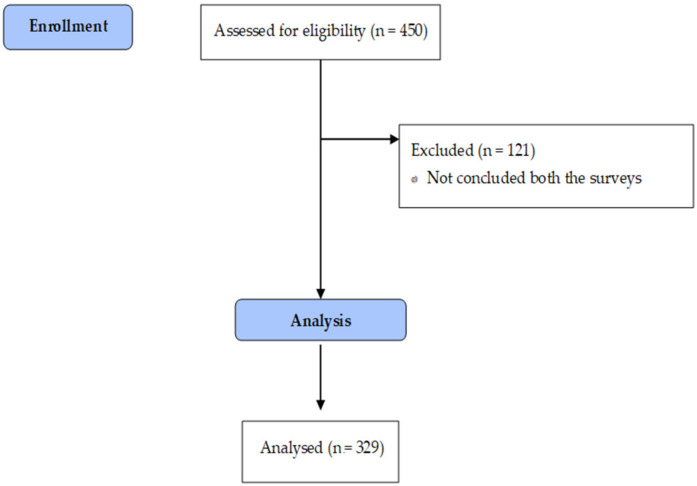
Participants’ flow-chart.

Figure 1 shows participants’ flow-chart. A total of 450 adults completed the questionnaire but a lot of participants did not complete both the surveys, so 121 were excluded from this analysis. Finally, 329 questionnaires were considered valid and evaluated.

### 3.2. Questionnaire

**Figure 2:**
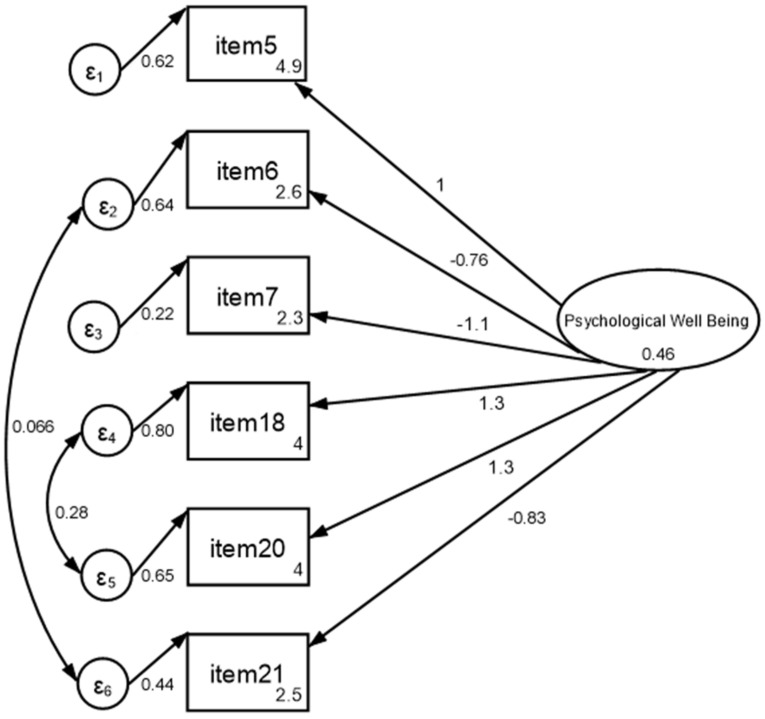
Path diagram of confirmatory factor analysis. Note: psychological wellbeing represents the latent variable, straight arrows represent paths whereas curved arrows covariances, values near each arrow represent the β coefficients of the model.

Figure 2 shows the path diagram resulting from the CFA, whereas Table 1 shows the fit statistics. The Cronbach’s alpha value was 0.845, which can be regarded as reliable. The model derived from the confirmatory factor analysis showed a fit with the data and all items explained 85% of the model variability. In addition, the chi-squared (χ^2^) test statistic was assessed for both model vs. saturated (χ^2^_(7)_ = 7.983, *p* = 0.33) and baseline vs. saturated (χ^2^_(15)_ = 756.5, *p* < 0.001). Finally, from the baseline comparisons, the comparative fit index (CFI) resulted as equal to 0.999, and the Tucker–Lewis index (TLI) result was 0.997; the standardized root mean square residual, SRMR, was 0.018.

### 3.3. Participant Characteristics

Table 2 shows participant’s main characteristics: the place of living, lifestyle habits and intention to practice physical activity. The majority of the participants in the study were females (78.1%); in females the youngest were less represented, while males’ sample was more homogeneous.

The youngest women who attended the activity came, in a considerable percentage, also from neighborhoods other than the one in which the park was located (59.5%); the participating women who live in the neighborhood increases with the increase in age classes, with significant differences between the age groups. In men, the percentage of participants coming from the neighborhood in which the park was located is highest in the age class >65 yrs, but without significant differences among age groups.

In both sexes, the most commonly used means of transport among people aged 18–44 yrs and >65 is by walk or by bike, while participants aged 45–64 yrs mostly used a car, motorcycle or scooter; the differences were significant only among females, both at baseline and at follow-up. Regarding the difference in the means of transport used at baseline and at follow-up, females presented a significant difference (χ^2^ = 27.4, *p* = 0.002), while males did not. In any case, it is worth emphasizing the increase in walking and cycling in males at follow-up.

The majority of people have reported the use of the stairs rather than the elevator. In addition, the frequencies of the use of stairs increased with age and were significant in males at follow-up. An increase in the use of stairs between the two measurements was observed, even if it was not significant. About one third of participants by age and sex declared to have started the practice of physical activity thanks to this project, with a smaller frequency in the youngest males; nevertheless, the differences among age classes were not significant.

Citizens of both sexes and of all the ages classed planned to continue to practice physical activity even after the end of the project, and the majority thought of practicing it with a frequency equal or greater to 2.5 h/week. The differences among age classes were not significant. In both sexes, height was higher in the youngest (Table 3) and BMI increased with the increase in age classes and did not show any significant differences between baseline and follow-up in all the age classes. Males generally presented a higher percentage of overweight and obese subjects than females, while females showed also underweight subjects. Analogously to BMI, weight status did not show any significant differences between baseline and follow-up (Table 3).

Table 4 shows participant’s health status and wellbeing.

At follow-up, the importance given to physical activity practice has increased, with significant differences among females in older age classes and in males in the youngest. All psychosocial parameters showed an improvement after following the three months physical activity program, with a reduction in states of tension, sadness and fatigue, and an improvement in the state of energy, serenity, and vitality. Significant differences were observed in females in all age classes, with few exceptions. Although, in males, the most significant differences are reported in the older age groups, in general, the differences found were not always significant (Table 4).

## 4. Discussion

The aims of the present study were to value the effectiveness of a structured PA intervention, administered to citizens in Bologna’s parks (Italy), on physical activity behaviors (intention to practice PA, importance of PA), and on weight status and psychological general wellbeing. A systematic review of interventions aiming to promote PA in urban green spaces has illustrated important gaps in the evidence: person based intervention constituted a very small number of studies overall, usually of low methodological quality [46]. In addition, to our knowledge, in the few studies that considered person based intervention, the PA prescription was delivered and not followed by expert technicians [36,37]. Thus, this study aimed to quantify the effect of a supervised and structured PA on the above mentioned parameters.

As regards the first aim of this study, it is noteworthy that about a third of the participants reported to have started the practice of physical activity thanks to this project. A high percentage of the participants (97–100% of the females; 95.5–100% of the males) planned to continue to practice physical activity even after the end of the project, and the majority thought to practice it with a frequency equal or greater to 2.5 h/week. These results strongly suggest the importance of involving qualified personnel. Trainers, indeed, can conduct the activities and encourage an increase in participation, by creating an environment of trustworthiness as well as continuity. In addition, trainers can also create and promote a program of events with clearly defined dates and places for local populations. The importance of supervised physical activity also emerges from two other studies [36,37], who valued the effectiveness of a park PA prescription intervention for improving total moderate to vigorous PA (MVPA) and other PA related behaviors, among adults, comparing participants in the intervention group, who received face to face counselling on PA with participants of the control group, who continued with their daily routine. Supervised PA resulted in meaningful and statistically significant increases in recreational PA, time spent in parks, and PA in parks, but did not improve psychological distress, accelerometer measured moderate to vigorous PA, and cardiometabolic outcomes. Our results confirmed, as in most of the studies [18,47], that urban green space plays an important role in promoting physical activity, especially among women and the elderly, improving awareness towards a real change in the active lifestyle. It should be noticed, as in other studies results, that women are more prone to physical activity because it is seen by many women as a way to relieve tension, to feel better, and to generate a healthy sense of fatigue [6].

The second aim was to investigate the effect of supervised PA on weight status and psychological general wellbeing. The PA intervention resulted in an improvement in all the selected wellbeing outcomes, with a reduction in the state of tension, sadness, fatigue, and an improvement in the state of energy, serenity, and vitality. The differences were more evident in females than in males. Green space is widely regarded as a health-promoting feature and has been linked to wellbeing, helping people to avoid the sense of isolation and, in this way, reduce the risk of depression and anxiety and improve the resilience and manageability of people. Higher levels of neighborhood green space have been associated with significantly lower levels of symptoms for depression, anxiety, and stress [48]. Being active in nature may be an important mechanism of the intervention effects on behavioral and quality of life outcomes [36]. Sellers et al. (2012) explored the effects of the environment on an individual’s PA by estimating differences between a 30-min self-timed brisk walk taken in a park compared with one taken in an urban setting in Glasgow, Scotland [38]. This study showed that the environment can impact healthy adults’ capacity to perform health-enhancing PA. Indeed, the park environment allows the individuals to walk briskly with fewer stops than in an urban environment [38]. In addition, de Bloom et al. (2017) found positive effects of the park-walking activities on workers’ recovery from work during lunch breaks [39]. The most consistent positive effects across the day were reported by the park walking group. Park walks and relaxation exercises during lunch breaks can enhance knowledge workers’ recovery from work, but the effects seem small in magnitude and rather short in duration. The results of the present study are in accordance with these results, suggesting that PA in a public park could be a potential strategy to improve the wellbeing of populations. These beneficial effects were seen over a medium term period (three months) and, in particular, in females in the different age groups.

While many studies have identified positive associations between urban green space and various aspects of individual health, the evidence linking green space to decreasing obesity rates remains equivocal. In the present study, no significant variations in BMI or weight status have been observed. According to Browning et al. (2017), the apparent conflict in existing evidence could be attributable to various methodological issues: the absence of objective measures of obesity in some studies, use of pool rather than individual data, or insufficient control for potentially confounding factors, and short term follow-up periods that cannot match the effects induced by aerobic exercise, considering the importance of a dosage administered with a progressive and long term principle to achieve this goal [49]. Even if, in the present study, the direct effects of the supervised PA on BMI did not emerge, it should be considered that the duration of the intervention was limited. However, the desired effect of a reduction in a sedentary lifestyle, capable of reducing weight imbalances in the long term, emerged from the intentions of the participants to continue to practice physical activity even after the end of the project.

Despite the contributions that the present study provides to the knowledge of the subject, some limitations and strengths deserve discussion. The questionnaire was the main data collection tool. The main criticisms raised concerning this approach are related to “nonobjectivity”, to the mixture between the object of study and the detecting, and to the degree of a priori knowledge of the population itself, not only on the size of the sample. Therefore, the interpretation of these associations as causal effects must be made with due caution. The strength of the study is undoubtedly the three-month follow-up, which is often absent in similar studies. This period was sufficient to highlight the increased value of the practice of physical activity with important findings in the change of an active lifestyle, with significant differences in females in the older age groups and males in the younger classes.

The perspectives above concern the reliability of the data that would require confirmation through quantitative research with experimental data. The modern phase of the development of technological systems for the sampling of biological and environmental parameters, and the processing of the data thus obtained for research purposes, began several years ago.

Thus, so called “smart technologies” can help us acquire valuable data with simple acquisition methods for the user, both for the observed person or the operator carrying out the observation. It is also possible to organize remote control protocols during the activity for the individuals to whom physical activity is administered to maintain performance and psychophysical integrity or in a clinical context. The wearable devices are equipped with sensors whose characteristics allow a new way of detecting biovital indicators and parameters of daily life activities, providing targeted analyses of the individual lifestyle. These devices have many advantages, including the acquisition of signals that occur in a noninvasive, prolonged, and personalized, also allowing a new ability to interrelate environments, activities, and behaviors. All this would allow greater reliability in the control of physical activity, both in terms of methodology and in the recording and acquisition of parameters useful for identifying the volume of work to be correlated with the effects induced in short, medium, and long term control groups. An important avenue for further research is also to increase knowledge aimed at understanding what types of green spaces matter and how they could be restored and redesigned to optimize the health and wellbeing of the population.

## 5. Conclusions

The aim of the present study was to assess the effectiveness of the project “The moving parks project”, which provides for the administration of supervised PA by qualified instructors to citizens within Bologna’s Park, on physical and psychosocial health and wellbeing. The results of this study are interesting, because, as far we know, there are few similar studies related to this aspect and no study that concerns Italy. There is an increasing interest to design interventions aimed at increasing PA in Italian adults and to understand their effects. The impact of the project “The moving parks” seemed to be positive and to represent a good strategy to improve health outcomes. It would be important to continue to propose this kind of project and to extend the initiative also to other cities.

## Figures and Tables

**Table 1:** CFA fit statistics.

Dependent Variables	Variance			R^2^	χ2	*p*	CFI	TLI	SRMR	Cronbach α
	Fitted	Predicted	Residual							
item 5	1.075	0.456	0.618	0.424						
item 6	0.897	0.261	0.635	0.291						
item 7	0.752	0.531	0.22	0.706						
item 18	1.522	0.721	0.8	0.474						
item 20	1.4	0.753	0.648	0.537						
item 21	0.752	0.312	0.44	0.414						
Model				0.85	7.983	0.33			0.018	0.845
Baseline					756.5	<0.001	0.999	0.997		

**Table 2:** Place of living, lifestyle habits and intention to practice PA of the participants.

	Females (257)					Males (72)				
	18–44 Yrs.	45–64 Yrs.	>65 Yrs.	Χ^2^	*p*	18–44 Yrs.	45–64 Yrs.	>65 Yrs.	Χ^2^	*p*
N (%)	42 (16.3)	115 (44.7)	100 (38.9)			21 (29.2)	24 (33.3)	27 (37.5)		
Live in the neighborhood (yes)	40.5	61.4	79.8	22.3	0.001	42.9	41.7	63.0	2.9	0.233
Way used for travel (baseline)										
Car, motorcycle, scooter	31.0	43.9	21.4	12.4	0.015	42.9	50.0	40.7	2.2	0.695
Walking or cycling	47.6	38.6	51.0			42.9	33.3	51.9		
Public transport	21.4	17.5	27.6			14.3	16.7	7.4		
Way used for travel (follow-up)										
Car, motorcycle, scooter	33.3	42.1	19.0	14.6	0.006	38.1	41.7	22.2	2.7	0.603
Walking or cycling	47.6	38.6	49.0			47.6	45.8	55.6		
Public transport	19.0	19.3	32.0			14.3	12.5	22.2		
Usually use (baseline)										
Elevators	28.2	33.0	41.8	2.7	0.254	27.8	30.0	41.7	1.1	0.582
Stairs	71.8	67.0	58.2			72.2	70.0	58.3		
Usually use (follow-up)										
Elevators	22.0	27.4	38.5	4.8	0.090	10.0	20.8	48.1	9.2	0.010
Stairs	78.0	72.6	61.5			90.0	79.2	51.9		
Start PA practice with the project (yes)	26.2	32.1	28.9	0.6	0.748	9.5	25.0	25.9	2.5	0.282
Plan to practice PA at the end of the project (yes)	100.0	98.2	97.0	1.4	0.490	100.0	95.5	96.2	0.9	0.631
Frequency with which participant intends to practice										
2.5 h	40.5	41.9	50.5	2.2	0.693	28.6	34.8	30.8	1.6	0.816
<2.5 h	16.7	16.2	11.6			19.0	13.0	7.7		
>2.5 h	42.9	41.9	37.9			52.4	52.2	61.5		

Note. Yrs. = years, PA = physical activity, χ^2^ = chi-squared, *p* = *p* value.

**Table 3:** Anthropometric parameters and weight status of the participants.

	18–44 Yrs.						45–64 Yrs.						>65 Yrs.			
	Mean	SD	Mean	SD	Z	*p*	Mean	SD	Mean	SD	Z	*p*	Mean	SD	Mean	SD
Females																
Height	164.5	6.2					163.6	6.4					159.1	5.6		
Weight	60.3	11.1	60.2	10.9	0.7	0.465	61.7	9.1	61.8	9.2	0.0	0.981	63.7	9.7	63.4	9.8
BMI	22.4	3.9	22.5	3.9	1.0	0.300	23.0	3.4	23.1	3.5	1.0	0.340	25.2	3.4	25.0	3.5
Males																
Height	177.7	7.7	177.9	8.4			175.6	7.3	175.3	7.4			174.7	5.8	175.0	6.3
Weight	73.7	10.8	73.6	10.8	0.4	0.674	75.7	11.0	76.1	10.4	1.4	0.154	74.5	7.9	74.8	8.6
BMI	23.5	3.5	23.4	3.7	0.1	0.889	24.6	3.5	24.8	3.6	0.0	1.000	24.4	2.6	24.4	2.6
Weight status	%		%		χ^2^	*p*	%		%		χ^2^	*p*	%		%	
Females																
Underweight	5.1		5.3		0.3	0.957	3.6		2.7		0.3	0.968	1.0		1.0	
Normal weight	76.9		76.3				73.0		73.2				53.5		55.2	
Overweight	10.3		13.2				19.8		19.6				36.4		35.4	
Obese	7.7		5.3				3.6		4.5				9.1		8.3	
Males																
Underweight	-		-				-		-				-		-	
Normal weight	75.0		76.2		0.5	0.785	70.8		65.2		0.4	0.804	61.5		61.5	
Overweight	20.0		14.3				25.0		26.1				38.5		38.5	
Obese	5.0		9.5				4.2		8.7				-		-	

Note. Yrs. = years, SD = standard deviation, Z = Z value, *p* = *p* value, χ2 = chi squared.

**Table 4:** Health status and well-being of the last 4 weeks from baseline to follow-up.

	18–44 Yrs.						45–64 Yrs.						>65 Yrs.						
	Mean Pre	(±SD)	Mean Post	(±SD)	Z	*p*	Mean Pre	(±SD)	Mean Post	(±SD)	Z	*p*	Mean Pre	(±SD)	Mean Post	(±SD)	Z	*p*	Range
Females																			
Importance of PA	71.6	(20.5)	76.4	(15.7)	1.8	0.068	73.1	(16.1)	77.1	(15.1)	3.4	0.001	73.3	(12.7)	76.4	(12.8)	2.7	0.007	0–100
Feel tense	4.5	(1.1)	5.1	(1.1)	2.6	0.010	4.8	(1.0)	5.2	(1.0)	3.1	0.002	5.1	(1.0)	5.4	(0.8)	2.6	0.010	1–6
Fell full of energy	2.7	(1.0)	1.9	(0.8)	3.6	0.000	2.6	(1.0)	2.2	(0.9)	3.8	0.000	2.6	(0.8)	2.2	(0.7)	3.7	0.000	1–6
Feel discouraged	2.5	(0.8)	2.0	(0.8)	2.9	0.004	2.3	(0.8)	2.1	(0.8)	2.4	0.017	2.3	(1.0)	2.0	(0.9)	2.5	0.012	1–6
Feel confident	4.0	(1.1)	4.4	(1.3)	2.0	0.049	4.0	(1.2)	4.2	(1.3)	1.4	0.173	4.1	(1.3)	4.5	(1.2)	3.1	0.002	1–6
Feel calm and happy	4.0	(1.2)	4.5	(1.1)	2.7	0.006	4.0	(1.1)	4.2	(1.1)	2.3	0.019	3.9	(1.2)	4.4	(1.1)	3.5	0.000	1–6
Feel tired	2.6	(0.9)	2.0	(0.6)	3.1	0.002	2.5	(0.8)	2.2	(0.8)	3.4	0.001	2.4	(0.9)	2.2	(0.8)	2.0	0.048	1–6
Males																			
Importance of PA	72.8	(16.9)	78.6	(16.6)	2.2	0.031	77.7	(16.3)	82.4	(15.1)	1.1	0.270	73.6	(11.6)	74.1	(12.4)	1.2	0.215	0–100
Feel tense	5.0	(1.3)	5.4	(0.6)	1.5	0.123	5.0	(1.0)	5.4	(0.8)	2.0	0.042	5.0	(0.8)	5.3	(0.5)	1.5	0.133	1–6
Fell full of energy	2.5	(1.4)	1.9	(0.9)	1.7	0.093	2.3	(0.8)	1.9	(0.5)	2.3	0.023	2.6	(0.8)	2.0	(0.8)	2.2	0.028	1–6
Feel discouraged	2.3	(1.1)	1.9	(0.7)	1.3	0.178	2.4	(0.8)	2.0	(0.9)	1.9	0.059	2.5	(0.8)	1.8	(0.6)	3.2	0.001	1–6
Feel confident	4.0	(1.5)	4.5	(1.3)	1.4	0.173	4.1	(1.4)	4.6	(1.0)	1.7	0.088	3.9	(1.2)	4.0	(1.2)	0.5	0.629	1–6
Feel calm and happy	4.3	(1.4)	4.5	(1.3)	0.6	0.529	4.3	(1.0)	4.4	(1.0)	0.6	0.569	3.6	(1.2)	4.3	(1.2)	2.9	0.003	1–6
Feel tired	2.4	(1.0)	2.1	(0.8)	1.2	0.249	2.6	(0.9)	1.9	(0.5)	2.6	0.009	2.3	(1.0)	1.9	(0.7)	2.0	0.041	1–6

Note. Yrs. = years, SD = standard deviation, Z = Z value, *p* = *p* value.

## Data Availability

Data may be requested from authors.

## Appendix A

The six parks are in different neighborhoods of Bologna and have different characteristics, listed below.

⯀ VELODROMO PARK

This park covers about 3 hectares. This park has several sports facilities, a children’s play area, green meadows and a bar. It also has an area equipped for physical activity, called “Ability Park”, in which anyone can train without barriers.

⯀ LUNETTA GAMBERINI PARK

This park covers about 14.5 hectares, it houses sports facilities, four schools, a community center, and a youth center. The main feature of the park is that it is surrounded by a thick mixed hedge that acts as a barrier against the traffic and noise of the surrounding streets. In addition, the park also has a play area for children, a skating rink and a dog area.

⯀ NICHOLAS GREEN PARK

This park covers about 8 hectares, has several play areas for children and a part dedicated to municipal vegetables gardens. In addition, it has wide lawns with pedestrian paths, but with few trees.

⯀ CEDRI PARK

This park covers about 11 hectares, it is characterized by large lawns, some woodland and a grove that runs along the river Savena, which defines the boundaries of the park. In the park, there is a wide plant variety, and, in addition, a children’s play area, benches, and fountains.

⯀ SAN DONNINO PARK

This park covers about 8 hectares. The park is located between the ring road and the railway and was born to redevelop the area. It is composed of large green areas, pedestrian paths, and rest areas. It also houses an educational garden and a wooden pavilion where there is an association of citizens who take care of the park itself.

⯀ VILLA ANGELETTI PARK

This park covers about 8.5 hectares, and is along the right bank of the canal Navile. In the park there is a long strip of natural vegetation that offers the possibility of naturalistic observations.
